# The Human Blood-Nerve Barrier Transcriptome

**DOI:** 10.1038/s41598-017-17475-y

**Published:** 2017-12-12

**Authors:** Steven P. Palladino, E. Scott Helton, Preti Jain, Chaoling Dong, Michael R. Crowley, David K. Crossman, Eroboghene E. Ubogu

**Affiliations:** 1 Neuromuscular Immunopathology Research Laboratory, Division of Neuromuscular Disease, Department of Neurology, University of Alabama at Birmingham, Birmingham, AL 35294-0017 United States of America; 2 Heflin Center for Genomic Science, Department of Genetics, University of Alabama at Birmingham, Birmingham, AL 35294-0024 United States of America

## Abstract

The blood-nerve barrier (BNB), formed by tight junction-forming microvessels within peripheral nerve endoneurium, exists to regulate its internal microenvironment essential for effective axonal signal transduction. Relatively little is known about the unique human BNB molecular composition. Such knowledge is crucial to comprehend the relationships between the systemic circulation and peripheral nerves in health, adaptations to intrinsic or extrinsic perturbations and alterations that may result in disease. We performed RNA-sequencing on cultured early- and late-passage adult primary human endoneurial endothelial cells and laser-capture microdissected endoneurial microvessels from four cryopreserved normal adult human sural nerves referenced to the Genome Reference Consortium Human Reference 37 genome browser, using predefined criteria guided by known transcript or protein expression *in vitro* and *in situ*. We identified 12881 common transcripts associated by 125 independent biological networks, defined as the normal adult BNB transcriptome, including a comprehensive array of transporters and specialized intercellular junctional complex components. These identified transcripts and their interacting networks provide insights into peripheral nerve microvascular morphogenesis, restrictive barrier formation, influx and efflux transporters with relevance to understanding peripheral nerve homeostasis and pharmacology, including targeted drug delivery and the mediators of leukocyte trafficking in peripheral nerves during normal immunosurveillance.

## Introduction

Peripheral nerves are structurally divided into three compartments: the outermost epineurium that consists of longitudinal arrays of collagen fibers in which fenestrated macrovessels directly derived from the extrinsic peripheral nerve blood supply reside (known as vasa nervorum); the inner perineurium, which consists of concentric layers of specialized myofibroblasts with intercellular tight junctions, that surround the innermost endoneurium, consisting of a loose array of collagen fibers in which myelinated and unmyelinated axons and non-fenestrated tight junction-forming microvessels reside, with rare leukocytes and fibroblasts^1–5^. In order to facilitate normal axonal signal transduction (essentially dependent on sodium/potassium flux) to and from the peripheral nervous system, the endoneurial microenvironment is strictly regulated^3,5,6^. Tight junction-forming perineurial myofibroblasts provide a critical interface between the endoneurial and epineurial interstitial fluid compartments that helps further regulate the endoneurial microenvironment; however, these cells are not in direct contact with circulating blood. Endoneurial microvessels are in direct contact with circulating blood, and are considered to form the blood-nerve barrier (BNB)^7^.

In mammals, the BNB is generally considered the second most restrictive mammalian microvascular barrier after the blood-brain barrier^6,8–12^. *In situ*, endoneurial microvessels share their basement membrane with pericytes that may act as specialized smooth muscle cells that regulate the microvasculature in response to differences in neural activity, blood pressure and volume^1,2,13^. Phenotypic and functional differences occur between micro- and macrovascular endothelial cells within the same tissue, between endothelial cells from different tissues and endothelial cells from the same tissues in different species, with molecular and functional adaptations occurring dependent on the cellular microenvironment^14–23^. Extrapolation of observations and functional mechanisms from other restrictive microvascular barriers, such as the blood-brain barrier, or epithelial cells, provides general knowledge on restrictive barrier systems that may not specifically apply to peripheral nerves.

There is limited knowledge on the molecular composition, and as a consequence, the mechanistic changes that occur at the human BNB during development, in healthy homeostatic states and in disease^4^. Such knowledge is paramount to understanding peripheral nerve angiogenesis, vascular differentiation and the role of the BNB in regulating the endoneurial microenvironment to facilitate normal axonal growth and myelination, as well as the development and maintenance of its specialized restrictive barrier and adaptations to physiological changes in the systemic circulation. Furthermore, transport of water, ions and polar molecules, solutes, nutrients (sugars, amino acids, fatty acids), macromolecules and drugs into and out of the endoneurium in physiologic and pathophysiologic states has direct relevance to peripheral nerve function in health and disease, including targeted drug delivery.

The BNB is a major interface of interaction between hematogenous leukocytes and peripheral nerve endoneurium, thus peripheral nerve immunosurveillance and adaptive or pathological alterations that facilitate leukocyte transmigration following injury, inflammation and in autoimmune neuropathies (i.e. peripheral nerve immunobiology) requires identification of the relevant molecular components^24^. Animal model studies aimed at understanding peripheral nerve leukocyte trafficking *in vivo* should be guided by knowledge derived from human observational *in situ* data in order to increase biological relevance and translational potential.

Significant advances have been made following the recent isolation and successful development of primary and immortalized human endoneurial or peripheral nerve endothelial cell lines that have resulted in some *in vitro* observational and functional studies relevant to understanding BNB angiogenesis, wound healing, response to extrinsic insult and metabolic derangements and alterations following physiological cytokine stimulus and normal/pathologic leukocyte trafficking^7,25–33^. However, current knowledge is incomplete as phenotypic changes may occur following primary endothelial cell isolation from endoneurial microvessels and culture compared to endoneurial endothelial cells *in vivo*, and immortalization may further alter the molecular composition and function of human BNB-forming endoneurial cells. Previous *in situ* peripheral nerve studies have identified several molecules expressed by the BNB using histochemical methods;^17,24,34–39^ however, such studies are limited by the quality of tissue preservation, availability and specificity of antibodies and other detection reagents, the clinical/scientific question being addressed, and possible ascertainment bias.

In order to more completely elucidate the molecular composition of the normal human BNB, as required to provide an essential framework to understand endoneurial endothelial cell biology and the molecular determinants of physiologically relevant interactions between the systemic circulation and peripheral nerve endoneurium, we performed whole transcriptome shotgun sequencing or RNA-Sequencing on cultured early (P3)- and late (P8)-passage adult primary human endoneurial endothelial cells (pHEndECs) and laser-capture microdissected endoneurial microvessels from 4 cryopreserved normal adult human sural nerves to determine transcripts conserved *in vitro* with progressive culture and expressed by BNB-forming endoneurial microvessels *in situ*. We validated the human BNB transcriptome *in situ* by indirect immunohistochemistry of 31 expressed proteins performed on a histologically normal cryopreserved adult sural nerve from an individual not used for RNA-sequencing.

## Results

### Sample description

**Figure 1:**
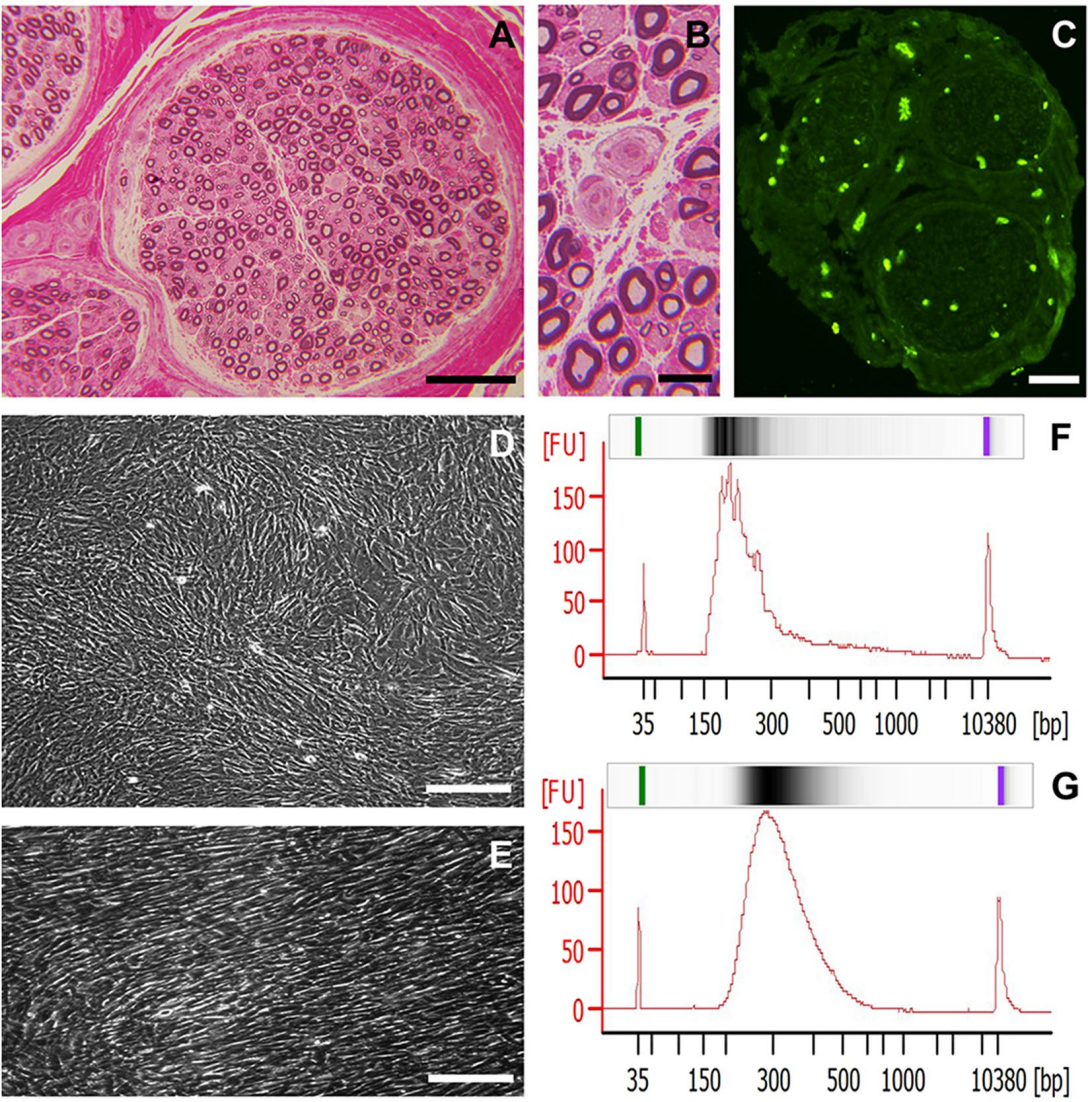
Sample characteristics. A representative digital light photomicrograph of a normal adult sural nerve plastic-embedded axial section, depicting the three peripheral nerve compartments is shown (**A**), with normal appearing endoneurial microvessels shown at higher magnification (**B**). A representative digital fluorescent photomicrograph of a normal sural nerve axial cryosection shows UEA-1-positive (green) endoneurial and epineurial vessels (**C**). A representative phase contrast digital photomicrograph shows confluent pHEndEC monolayers in culture (**D**), with a higher magnification image demonstrating their spindle shape without intercellular gaps (**E**). High sensitivity digital electropherograms following ribosomal RNA depletion demonstrate high quality cDNA for RNA sequencing from laser-capture microdissected endoneurial microvessels from a single normal adult (**F**) and P3 pHEndECs (**G**). Scale bars: A = 100 µm, B = 10 µm, C = 500 µm, D = 200 µm, E = 100 µm.

Archival slides of 1% toluidine blue-stained, basic fuchsin-counterstained 1 μm thick semi-thin plastic-embedded sections from the four normal adult sural nerves (2 men and 2 women, mean age 51 years at the time of biopsy) demonstrated normal axonal density (Fig. 1 A) and normal appearing endoneurial microvessels (Fig. 1B). FITC-conjugated Ulex Europaeus agglutinin-1 (UEA-1, a lectin that specifically binds to α-fucose residues and is generally considered to be the best marker of human vascular endothelial cells)^7,40^ accurately identified endoneurial microvessels and epineurial macrovessels on Optimum Cutting Temperature (OCT)-preserved axial sural nerve cryostat sections (Fig. 1C). The minimum total endoneurial microvessel area dissected per sural nerve biopsy was 135,516 μm^2^. Phase contrast photomicrographs demonstrate the spindle-shaped pHEndEC monolayer in culture 24 hours after confluence (Fig. 1D and E). Following reverse transcription, High Sensitivity cDNA electropherogram traces following depletion of ribosomal cDNA confirmed library quality prior to next generation sequencing (>90% between 150–750 base pairs in size, with >75% between 200–400 base pairs), as shown for a representative laser-capture microdissected pooled and concentrated endoneurial microvessel sample (Fig. 1F) and the P3 pHEndEC sample (Fig. 1G). The minimum concentration of cDNA obtained was 1900 pg/μL for endoneurial microvessels, with an average of 3100 pg/ μL obtained from confluent pHEndECs.

### Sequenced transcript quality and human genome alignment

Quality (Q) Scores demonstrated a probability of base calling error for each base pair by the sequencer for each sample as <0.001 for all samples, negating the need to remove any low quality reads/bases from the datasets. cDNA from all 4 laser-capture microdissected endoneurial microvessel samples had >50% of sequenced reads align uniquely or to multiple loci of the human genome (range 50.62–85.46%) with average input read length 137 (range 123–145). P3 and P8 pHEndEC cDNA had >95% of sequenced reads align uniquely or to multiple loci of the human genome (P3 96.67%; P8 96.72%) with an input read length of 98 for both samples. For all samples, >20 million reads aligned to the human reference genome (range 22.3–40.6 million), with no chimeric reads detected.

### Human BNB transcriptome

Using predefined criteria of Fragments Per Kilobase of transcript per Million (FPKM) >0.1 (based on the lowest observed value of identified transcripts previously known to be expressed by the human BNB *in vitro* and *in situ*), we identified 18002 transcripts from a total of 60252 transcripts expressed by both P3 and P8 pHEndECs (the *in vitro* BNB [IVBNB]), with an 80.2% transcript overlap between these passages. 2122 transcripts were identified in the P3 pHEndECs only, associated by a total of 40 independent networks and subnetworks that predominantly reflected regulation of cell division and gene transcription (Supplementary Data file S1). 2312 transcripts were identified in the P8 pHEndECs only, associated by a total of 109 independent networks and subnetworks that predominantly reflected endothelial cell de-differentiation (83 independent networks; Supplementary Data file S2). A total of 15375 transcripts (out of 60252) were expressed by at least three of four laser capture microdissected microvessels (the *in situ* BNB [ISBNB]).

**Figure 2:**
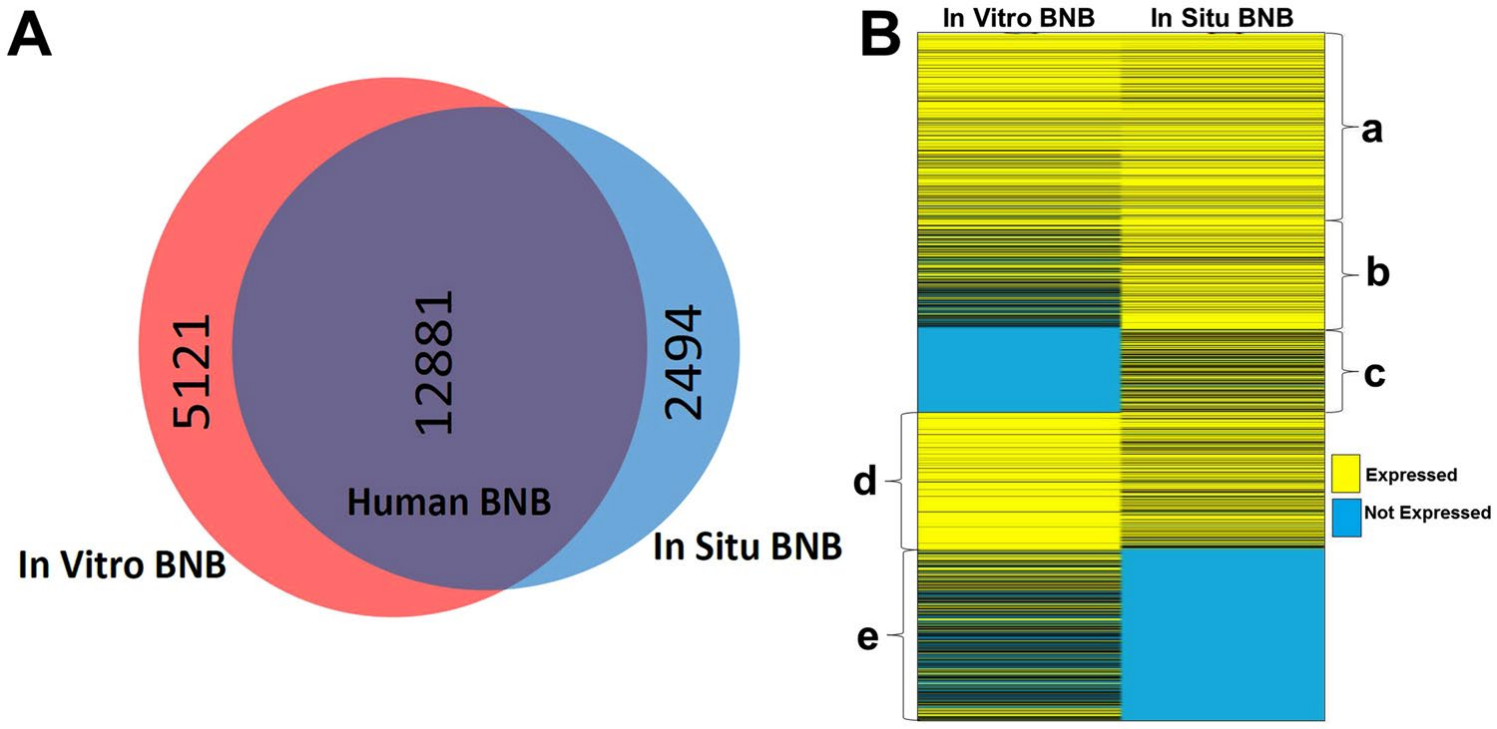
Human BNB transcriptome. Venn diagrams depict the human BNB defined as transcripts expressed by both the *In Vitro* BNB and *In Situ* BNB, with numbers indicating the number of transcripts identified based on predefined criteria (**A**). Hierarchical clustering (**B**) demonstrates transcripts that are equally expressed *in vitro* and *in situ* (**a**), highly expressed *in situ* (**b**), expressed *in situ* only (**c**), highly expressed *in vitro* (**d**) and expressed *in vitro* only (**e**).

We identified 11281 transcripts expressed by the IVBNB and ISBNB, defining the normal human BNB transcriptome. These transcripts were associated by a total of 125 independent biological process, cellular component and molecular function networks, encompassing a total of 1369 networks and subnetworks (Supplementary Data file S3). There was a 67% transcript overlap between deduced human BNB and IVBNB, with an 84% overlap between the deduced human BNB and ISBNB, with an additional 5121 transcripts expressed *in vitro* only and 2492 transcripts expressed *in situ* only (Fig. 2A). Hierarchical clustering further demonstrated similarities (i.e. the human BNB) and differences between the IVBNB and ISBNB (Fig. 2B).

The transcripts expressed *in vitro* only were associated by 20 independent biological networks (total of 31 networks and subnetworks) that are predominantly indicative of active cell division, but also indicate cytokine/chemokine binding and regulation of leukocyte trafficking networks not detected *in situ* (Supplementary Data file S4). The transcripts expressed *in situ* only were associated by 42 major independent networks (total of 192 networks and subnetworks) that include myelination, nervous system development, glial cell differentiation, neuronal action potential signaling and circulatory system processes. These networks implied significant exclusion of *in situ* contaminating Schwann cells, axons and pericytes from the deduced human BNB transcriptome (Supplementary Data file S5).

**Figure 3:**
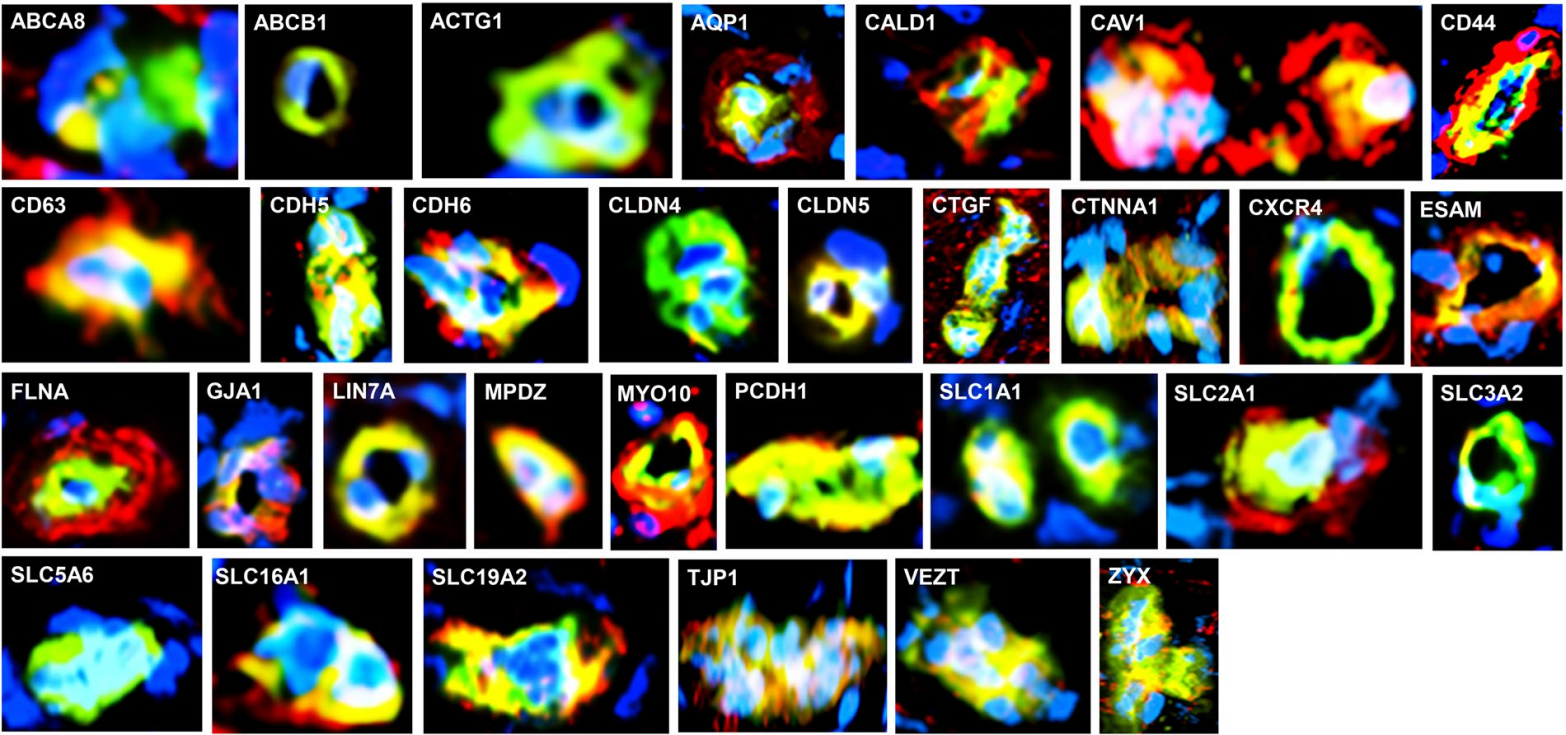
Validation of the human BNB transcriptome. Merged digital photomicrographs of sural nerve endoneurial microvessels (UEA-1 FITC-positive, green) *in situ* show BNB endothelial cell expression (yellow-green, yellow or orange co-localization dependent of the relative fluorescent intensity of protein marker in red) of transporters (*ABCA8*, *ABCB1*, *AQP1*, *SLC1A1*, *SLC2A1*, *SLC3A2*, *SLC5A6*, *SLC16A1* and *SLC19A2*), cytoskeletal proteins (*ACTG1*, *CALD1*, *FLNA*, *MYO10*), cell membrane proteins (*CAV1*, *CD44*, *CD63*, *ESAM*), junctional complex proteins (*CDH5*, *CDH6*, *CLDN4*, *CLDN5*, *CTNNA1*, *GJA1*, *LIN7A*, *MPDZ*, *PCDH1*, *TJP1*, *VEZT*, *ZYX*), vascular endothelial cell secreted protein *CTGF*, and chemokine receptor *CXCR4*. Positive expression of *AQP1*, *CALD1*, *CAV1*, *CD44*, *CD63*, *FLNA*, *MYO10* and *SLC2A1* (red) by cells that surround and are in direct contact with endothelial cells (most likely pericytes), and *ABCA8* by cells close to but not in direct contact with endothelial microvessels (most likely Schwann cells) are also observed. Nuclei are identified in blue. Original magnification 400X.

The deduced human BNB transcriptome included previously identified vascular endothelial markers, enzymes, scavenger receptors, mitogen receptors, nutrient transporters, cellular adhesion molecules, chemokines, adherens and tight junction and junction associated molecules^4,7,17,24,27,28,32,35,37,41^, validating the accuracy of the deduced transcriptome. These had been identified by polymerase chain reaction (PCR), immunohistochemistry or western blot. We further validated the BNB transcriptome by demonstrating expression of 31 selected cell membrane, chemokine receptor, cytoskeletal, junctional complex and secreted proteins by endoneurial microvessels *in situ* by indirect fluorescent immunohistochemistry (Fig. 3). The human BNB transcriptome also includes the uniformly expressed microvascular endothelial cell-specific transcription factor orthologs previously identified in mice^42^. Biologically important networks identified of particular importance to a restrictive microvascular barrier include endothelial cell migration/differentiation/angiogenesis/morphogenesis, intercellular protein organization/assembly, regulation of molecular transport, and immune response processes.

A total of 408 transcripts expressed *in situ* were also expressed by P3 pHEndECs only. These transcripts were only associated by 4 independent biological networks: single organism signaling, nervous system development, cell communication and cell periphery; supporting the notion that these transcripts predominantly represent contaminating Schwann cell and axonal transcripts that were lost with endothelial cell culture (Supplementary Data file S6). However, glial-derived neurotrophic factor (GDNF) receptor *GFRA1* (FPKM P3 = 0.15, P8 = 0) and its tyrosine kinase component, *RET* (FPKM P3 = 0.17, P8 = 0), previously detected and biologically active in response to exogenous GDNF at the human BNB *in vitro* during restoration of restrictive barrier characteristics following diffuse injury^27^, were detected through this analysis. Other potentially relevant BNB transcripts that may have been lost with pHEndEC culture based on our detection criteria include *ABCB4* (energy-dependent phospholipid efflux transporter, FPKM P3 = 0.62, P8 = 0.05), *AQP7* (water channel, FPKM P3 = 0.28, P8 = 0), *CDH19* (adherens junction protein, FPKM P3 = 1.99, P8 = 0), *CELSR2* (adherens junction protein, FPKM P3 = 0.41, P8 = 0.09), *CGN* (tight junction associated protein, FPKM P3 = 0.14, P8 = 0), *GJC3* (gap junction protein, FPKM P3 = 0.16, P8 = 0.09), *SLC17A7* (sodium-dependent phosphate transporter, FPKM P3 = 0.3, P8 = 0.09), *SLC1A3* (glutamate transporter, FPKM P3 = 0.63, P8 = 0.09), SLC22A15 (organic ion transporter, FPKM P3 = 0.14, P8 = 0.01), *SLC35F1* (unknown function, FPKM P3 = 1.34, P8 = 0.04), *SLC38A3* (sodium-coupled neutral amino acid transporter, FPKM P3 = 0.71, P8 = 0.08), and protocadherins, *PCDHGC4* (FPKM P3 = 0.17, P8 = 0.08) and *PCDHGC5* (FPKM P3 = 0.21, P8 = 0.03).

**Figure 4:**
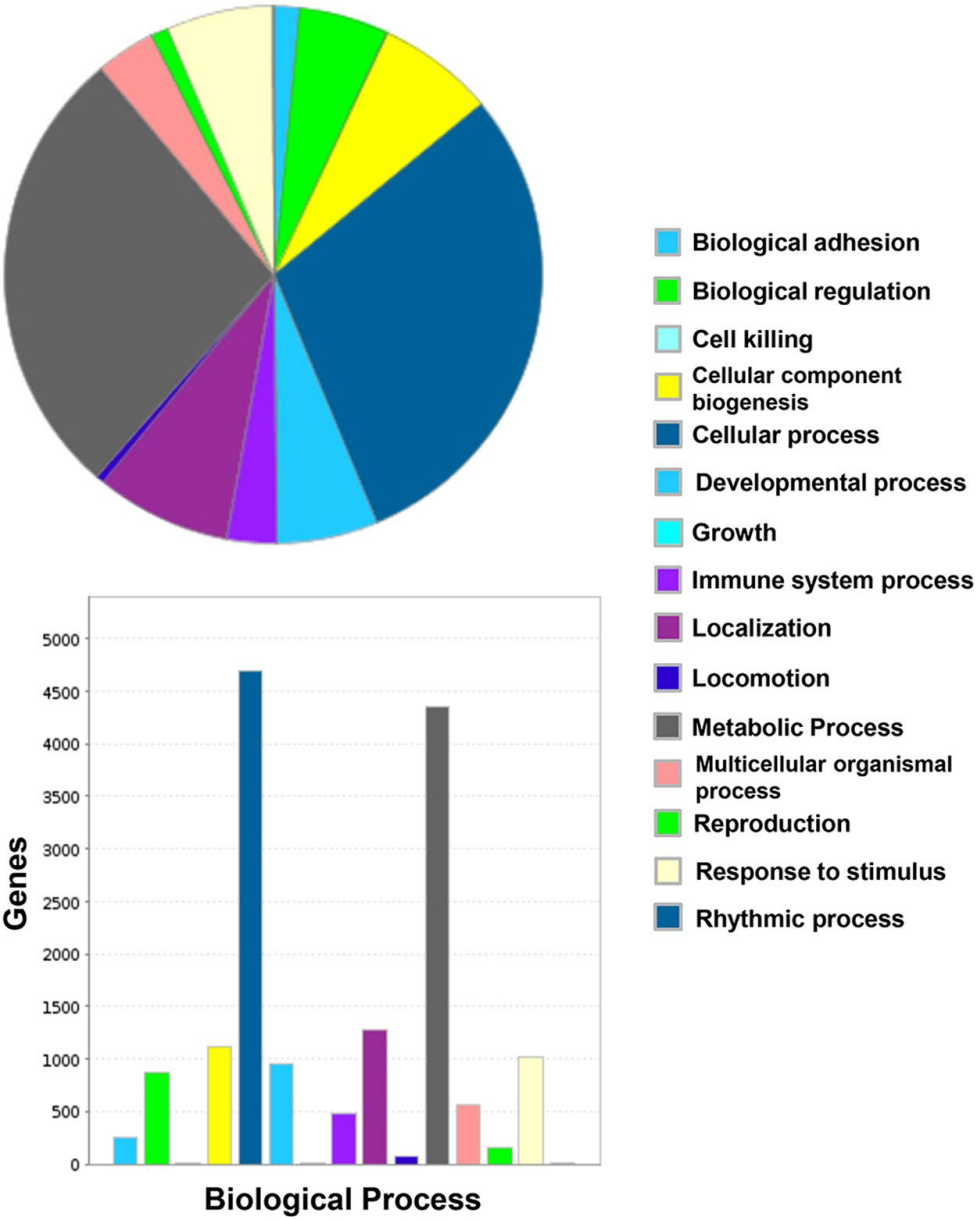
Human BNB Biological Process. PANTHER generated pie charts and bar histograms demonstrate the diversity of human BNB biologic processes and the number of genes implicated in each process.

**Figure 5:**
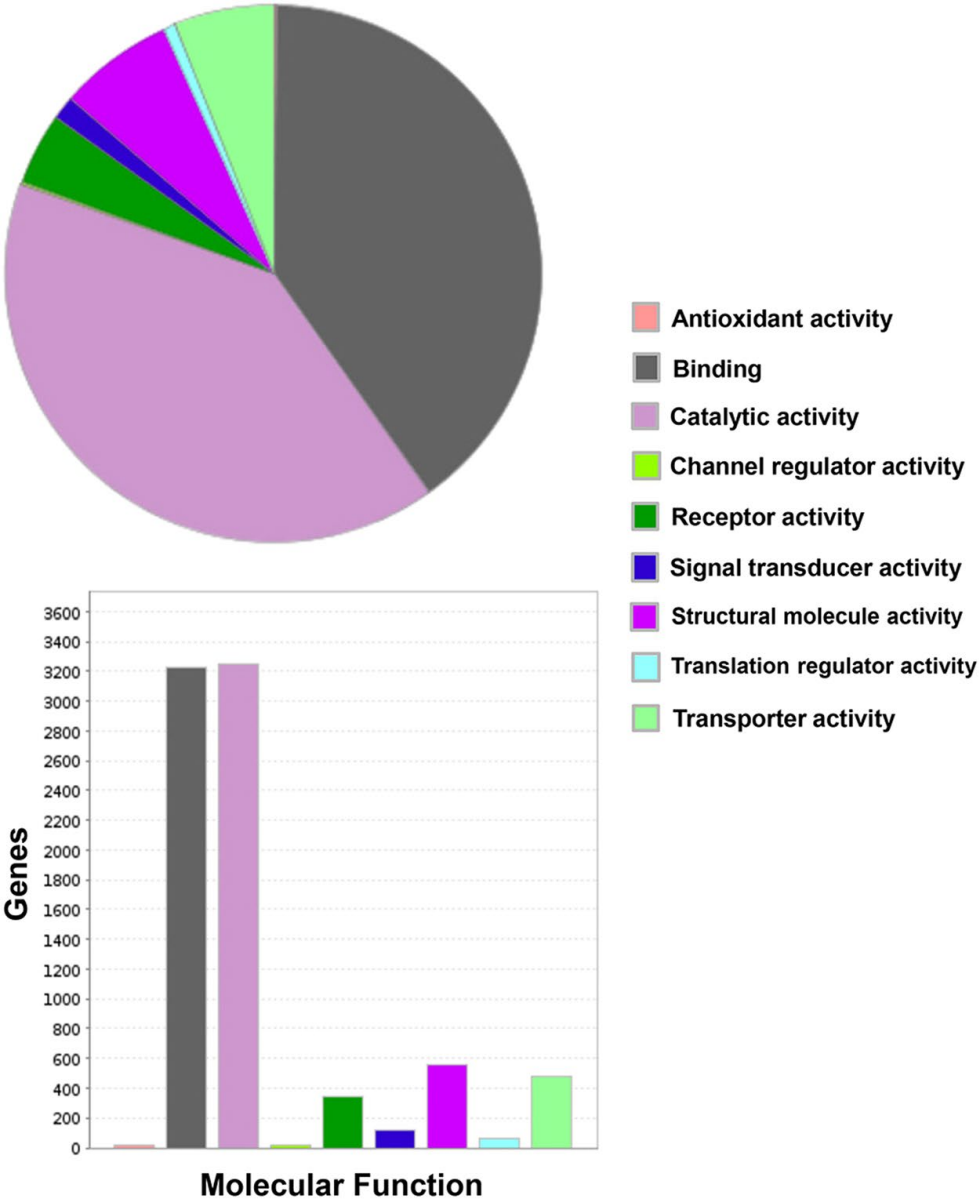
Human BNB Molecular Function. PANTHER generated pie charts and bar histograms demonstrate human BNB molecular function and the number of genes involved with each function.

**Figure 6:**
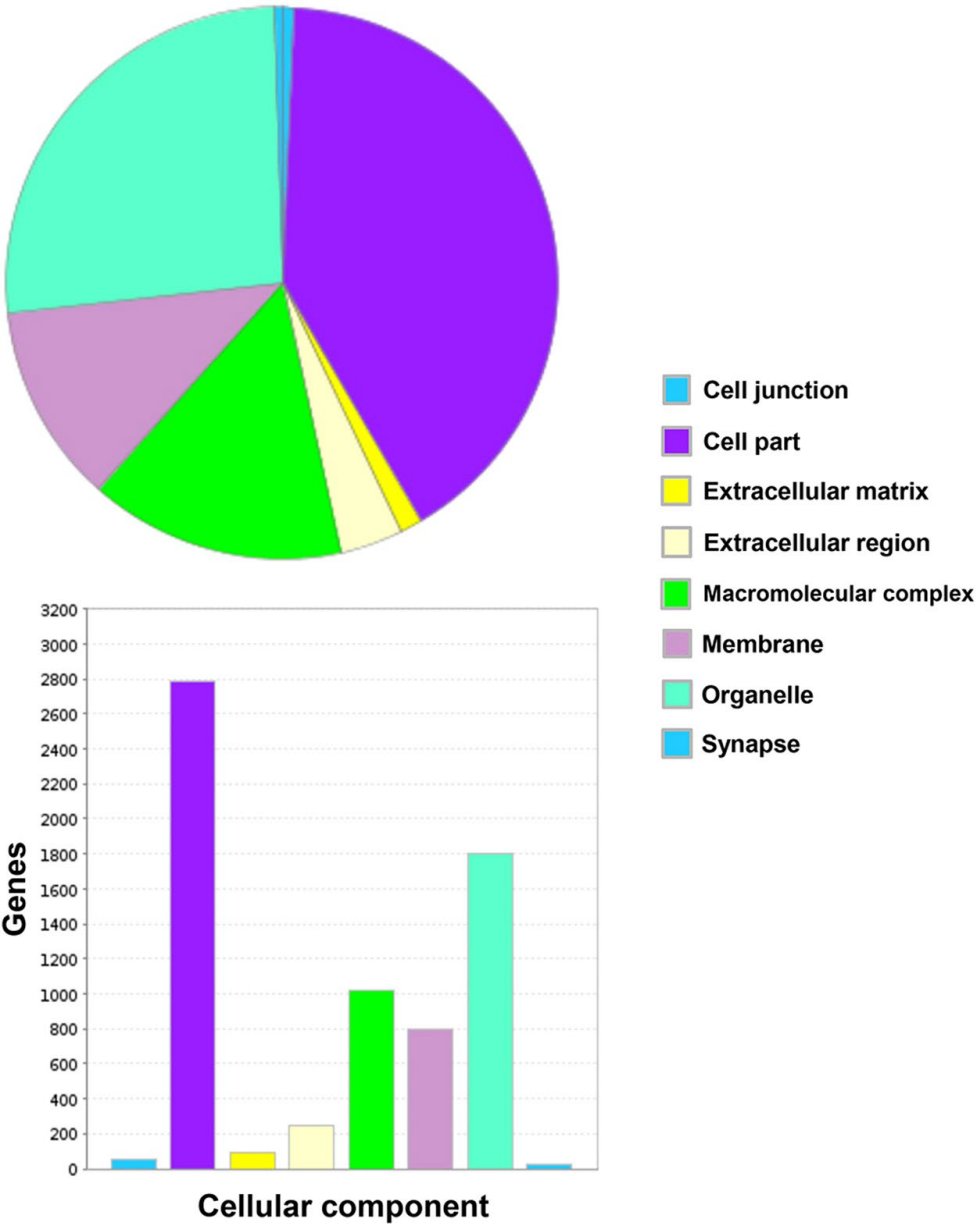
Human BNB Cellular Component. PANTHER generated pie charts and bar histograms demonstrate human BNB cellular component and the number of genes that contribute to each component.

**Figure 7:**
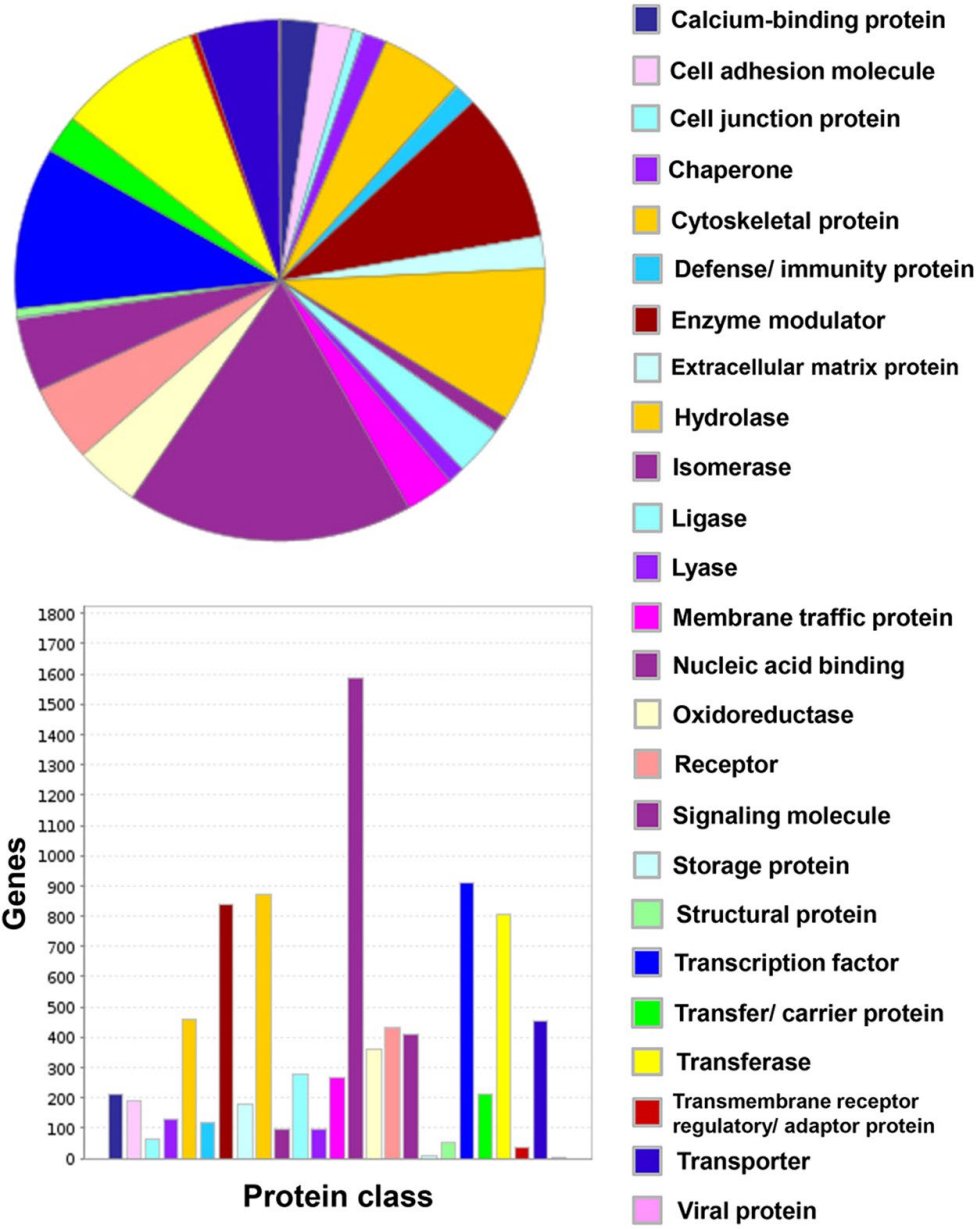
Human BNB Protein Class. PANTHER generated pie charts and bar histograms demonstrate the diversity of human BNB protein classes and the number of genes classified in each group.

There is a diverse repertoire of transcripts expressed by the deduced normal human BNB, supportive of an active role in homeostatic peripheral nerve function, and dispelling the notion that microvascular endothelial cells provide a relatively passive conduit for blood flow in normal healthy tissues. The repertoire of biological processes is depicted in Fig. 4, with “cellular process”, “metabolic process” and “localization” being three most common biological processes observed. In terms of molecular function, “binding”, “catalytic activity” and “structural molecule activity” represent the three most prevalent seen at the human BNB, as shown in Fig. 5, while genes involved in “cell part”, “organelle” and “macromolecular complex” are the most prevalent in terms of cellular component are shown in Fig. 6. The diversity of classified human BNB protein-coding transcripts is shown in Fig. 7, with “nucleic acid binding”, “transcription factor”, “hydrolase”, “enzyme modulator” and “transferase” being the most prevalent.

### Human BNB transporters

The human BNB demonstrated 509 transporter transcripts that include members of the solute carrier transporter family (196), ATP-binding cassette transporter family (33), cation channels (76), anion channels (13), zinc transporters (14), solute carrier organic anion transporters (4) and aquaporins (3), as shown in Supplementary Data file S7. The extensive array of influx and efflux transporters, including several amino acid, carbohydrate, monocarboxylate and multidrug resistant transporters previously or currently demonstrated at the BNB by PCR, immunohistochemistry or western blot *in vitro* or *in situ* ^4,7,25,30,37,43^, provides a blueprint to determine which transporters are required for human endoneurial endothelial cell metabolism, as well as those responsible for maintaining endoneurial homeostasis by transporting essential ions, polar molecules, solutes, nutrients and macromolecules from circulating blood into the endoneurium for normal Schwann cell and axonal function, and removing metabolic waste products and xenobiotics from the endoneurium.

### Human BNB intercellular junction complex

The molecular composition of the intercellular junction complex provides insights to the components of a specialized restrictive microvascular barrier system in health, as required to determine alterations that may occur in disease states. We ascertained expression of 133 intercellular junctional complex transcripts, including 22 tight junction or junction-associated molecules, 45 adherens junction or junction-associated molecules, and 52 cell junction-associated or adaptor molecules by the human BNB (Supplementary Data file S8), significantly expanding previous limited knowledge^4,5,7,27,30,32,35^. Tight junction transcripts include 6 members of the claudin family (*CLDN1*, *CLDN11*, *CLDN12*, *CLDN15*, *CLDN4* and *CLDN5*), occludin, zona occludens-1 and -2, junction adhesion molecules-1, -2 and -3, and endothelial cell adhesion molecule. Adherens junction transcripts include cadherins (*CDH11*, *CDH13*, *CDH15*, *CDH2*, *CDH24*, *CDH5* and *CDH6*) and 36 protocadherins (a cadherin subfamily highly expressed in neural tissues)^44^. Cell junction-associated or adapter transcripts include the catenin family (α1, β1, δ1, and γ), nectin cell adhesion molecule-1, -2 and -3, and 8 members of the membrane associated guanylate kinase family. Five gap junction channel transcripts (known to facilitate intercellular communication [including between endothelial cells], as may be required for autocrine signaling pathways, for example, during neurodevelopment^45^) were also detected (*GJA1*, *GJA4*, *GJA5*, *GJC1*, *GJC2*).

### Chemokines and chemokine receptors

We determined which chemokines and chemokine receptors may have homeostatic roles during immunosurveillance at the human BNB or actively participate in innate immune responses. These include CCL14 (induces enzyme release from monocytes), CCL2 (monocyte/macrophage and T-cell trafficking), CX3CL1 (natural killer, monocyte/macrophage, and T-cell trafficking), CXCL16 (natural killer T-cell trafficking and survival), and CXCL3 (neutrophil trafficking)^46^. Expression of CXCL12 with its signaling receptor CXCR4 and atypical receptor ACKR3 (also known as CXCR7) that serves to modulate chemokine gradients for CXCR4, implies an important autocrine effect at the human BNB, such as in vascular remodeling^46^. Interestingly, expression of CCL28 and one of its receptors, CCR10 by the human BNB similarly implies an autocrine role in normal endoneurial endothelial cell physiology (e.g. endothelial cell migration and capillary formation)^47^, suggesting redundancy in an important microvascular biological process. CXCR5 (typically expressed by B- and T-cells and has been recently shown to facilitate extracellular matrix remodeling)^48^ is also expressed by the human BNB.

As  stated previously, chemokine binding and regulation of leukocyte trafficking networks were detected *in vitro* that were not observed *in situ*. Transcripts detected *in vitro* only include chemokines involved in neutrophil trafficking (CXCL1, CXCL2, CXCL5, CXCL6 and CXCL8), T-helper 1 cell trafficking (CXCL9, CXCL10, CXCL11), T-helper 2 cell trafficking (CCL7, CCL8 and CCL26), T-helper 17 trafficking (CCL20), T-cell, and monocyte/macrophage trafficking as part of the innate and adaptive immune responses (CCL3, CCL4 and CCL5) and CCL16 implicated in dendritic cell maturation^46^. Atypical chemokine receptor CCRL2 (also known as ACKR5; function is currently undefined, but may serve as a “chemokine sink”) is also expressed by the IVBNB. It is possible that these endothelial cells are activated by *in vitro* culture resulting in increased chemokine gene transcription or there is some chemokine system dysregulation *in situ* following nerve biopsy and cryopreservation precluding detection and inclusion as part of the normal human BNB. Nonetheless, these data support an active role of the human BNB in hematogenous leukocyte interactions in peripheral nerves, as previously implicated^24,26,43,49,50^.

### Immunoglobulin transport

Of further clinical relevance is the transport of circulating immunoglobulins (pathogenic or therapeutic) across the tight junction-forming human BNB. We observed transcript expression of FCGRT (F_c_ fragment of IgG receptor and transporter) which binds to the monomeric region of IgG to facilitate its transcytosis to the apical or basolateral membrane of endothelial cells^51^ by the human BNB. However, PIGR (polymeric immunoglobulin receptor: binds dimeric IgA and pentameric IgM)^51^ was not expressed, suggesting that these large molecular weight antibody subclasses do not undergo human BNB transport in normal nerves or do so via undefined non-receptor-mediated transcytosis pathways. It remains to be determined whether adaptive or pathologic changes in FCGRT or PIGR expression occur at the human BNB with different disease states to modulate immunoglobulin transport between the blood circulation and the endoneurium.

## Discussion

We utilized RNA-sequencing on confluent P3 and P8 pHEndEC monolayers and normal adult laser-capture microdissected endoneurial microvessels to deduce the human BNB transcriptome. Genome Reference Consortium Human Reference 37 (hg19) was used rather than the more recently released Reference 38 (hg38) as hg19 is currently the most widely accepted genome to align human data to, with most of the current human genome databases utilizing hg19 coordinates.

pHEndECs isolated and purified from a single donor were used, and previous work demonstrated a ~95% purity at P3, using indirect fluorescent immunohistochemistry and flow cytometry, with loss of contaminating pericytes and fibroblasts with continued culture using our defined culture conditions that involved seeding these endothelial cells at 10,000–15,000 cells/cm^2^ ^7^. Variations in pHEndEC shape and diameter with differences in cytoplasm-to-nucleus ratio in culture had been observed based on the proliferating state^7^. By selecting transcripts expressed by both P3 and P8 pHEndECs to define the IVBNB transcriptome, we sought to identify transcripts that persisted with continuous cell culture and eliminate induced transcript expression as a direct consequence of recent endothelial cell isolation from peripheral nerves that required *ex vivo* manual endoneurial bundle stripping, aggressive enzymatic digestion and density gradient centrifugation, as well as the effects of pHEndEC de-differentiation that may occur as a consequence of extended *in vitro* culture.

By selecting transcripts expressed in endoneurial microvessels from three of the four normal adult sural nerves, we sought to include conserved transcripts in unrelated individuals and reduce the effect of contaminating luminal or migrating leukocytes, pericytes that share a basement membrane with endothelial cells and myelinated axons that are commonly reside ~5–10 μm away from endoneurial microvessels as shown on glutaraldehyde-fixed, osmium tetroxide post-fixed, plastic-embedded sections (Fig. 1B). A high concentration of UEA-1 FITC was used to identify endoneurial microvessels in ethanol-fixed cryostat sections at lower magnification to facilitate rapid laser capture microdissection as to limit RNA degradation associated with sample processing and direct laser effects. Rather than view and image the residual endoneurium following endoneurial microdissection, we observed each microdissection cap at higher magnification for extraneous material and excised these using tape or laser ablation, depending on its size.

Taking into account our definition of the human BNB transcriptome, the probability that transcripts identified as the IVBNB (18002/60252) and ISBNB (15375/60252) occur in both independently derived transcriptomes is 0.076 [(18002/60252) x (15375/60252)], supporting our notion that the identified transcripts are highly conserved by normal adult endoneurial endothelial cells *in vitro* and *in situ*. The validity of approach is further evidenced by the inclusion of transcripts that were previously demonstrated at the BNB *in vitro* or *in situ* by PCR, immunohistochemistry or western blot, our indirect fluorescent immunohistochemistry validation of endoneurial microvessel protein expression *in situ*, evaluating transcripts with mean FPKM values between 0.8 and 750.5 *in situ* and 0.4 and 3012.4 *in vitro* using another histologically normal adult cryopreserved sural nerve biopsy, as well as uniformly expressed microvascular endothelial specific transcription factor orthologs (i.e. vascular identity factors) previously observed in mice, including cerebral microvascular endothelial cells^4,7,17,24,25,28,30,35,37,42^. We also identified a few additional transcripts, such as the previously validated *GFRA1* and its receptor tyrosine kinase *RET* ^27^, intercellular junctional complex molecules and transporters that were expressed by laser-capture microdissected endoneurial microvessels *in situ* that were lost with pHEndEC culture. It is important to recognize that some “classic” markers of pericytes, Schwann cells and fibroblasts are known to be expressed by vascular endothelial cells, emphasizing the importance of using biological networks and subnetworks deduced from PANTHER analysis to aid identify cell types based on known or expected biological function^52–55^.

This comprehensive list of transcripts and the associated networks provides the framework for biologically relevant studies on human BNB angiogenesis during peripheral nerve development and following injury, formation of restrictive intercellular junctions and adaptations under normal physiological and pathophysiological states, hydraulic conductivity (i.e. transendothelial water flux) in response to changes in hydrostatic pressure and endoneurial interstitial volume and the transport kinetics of ions, polar molecules, solutes, nutrients, macromolecules (such as immunoglobulins) and drugs/ xenobiotics into and out of the peripheral nerve endoneurium in health and disease. Hypothesis-driven studies relevant to leukocyte-endothelial interactions unique to the human BNB during immunosurveillance would be guided by the established transcriptome profile of chemokines and other constitutively expressed cellular adhesion molecules. Furthermore, direct transcript database comparison of the human BNB transcriptome with restrictive and non-restrictive barrier-forming human microvascular endothelial cells, including the blood-brain barrier, would aid determine transcripts and specific biological networks potentially unique to the human BNB for functional assessment using pHEndECs *in vitro*.

Our overall strategy makes the assumption that any rare contaminating Schwann cells, pericytes, axons and endoneurial macrophages and fibroblasts present following pHEndEC isolation that may exist in early passage cultures would be lost with continuous culture in endothelial cell growth supporting conditions. Our data analysis showed 89.4% of transcripts expressed by P3 pHEndECs and 88.6% of transcripts expressed by P8 pHEndECs were expressed by both and included in the IVBNB transcript database, attesting to the fact that there was relatively little change in transcript expression between passages 3 and 8. This observation does not support preferential proliferation of contaminating cells such as pericytes with extended pHEndEC culture. Excluded transcripts were predominantly associated by networks involved in regulation of cell division and gene transcription (P3) and de-differentiation (P8).

We cannot exclude the possibility that cultured pHEndECs could have acquired some non-vascular transcripts due to early de-differentiation that overlap with these contaminating cells *in situ*, resulting in their inclusion as part of the human BNB transcriptome. *In situ* RNA degradation in OCT-cryopreserved sural nerves and subsequent degradation following endoneurial laser capture microdissection could have occurred, resulting in a failure to completely identify transcripts expressed by purified viable endoneurial endothelial cells *in vitro*. Another question is whether there is appreciable microvascular endothelial cell heterogeneity within the same or different parts of the peripheral nerve endoneurium. The sural nerve is one of the terminal branches of the sciatic nerve with its fascicles in direct continuation, so we do not necessarily expect differences in BNB molecular composition. We did not attempt to evaluate BNB diversity between normal adult nerves in this study; however deduction of the normal human adult BNB transcriptome provides a framework for future comparative studies. It is also quite possible that some of the expressed protein coding transcripts are not translated, or expressed in the presumed cellular compartments at the human BNB *in vivo*. The overall aim was to establish a comprehensive molecular signature of the normal human BNB, providing an essential blueprint or reference guide for future translationally relevant peripheral nerve biology studies in health and disease.

Targeting drug delivery across restrictive tight junction-forming microvascular barriers, such as the blood-brain barrier, and target site tissue retention at physiologically active concentrations have become essential considerations in therapeutic drug design strategies. This has implications for effective local and systemic drug administration targeting normal (e.g. anesthesia) and abnormal peripheral nerves (e.g. analgesia) with limited off-target or unwanted side effects. Knowledge of the components and regulators of small molecule and macromolecular transport unique to the human BNB would aid medicinal chemists and pharmacologists develop drugs with high BNB influx permeability, utilizing the array of influx transporters, channels and receptor-mediated transcytosis components to aid drug design, with specific modifications made to modulate drug efflux in the opposite direction as a means of increasing peripheral nerve endoneurial bioavailability and efficacy.

Detailed knowledge of the molecules relevant for BNB angiogenesis and intercellular junction complex formation could guide therapeutic strategies for peripheral nerve repair following traumatic injury, recognizing that rapid restoration of the normal endoneurial microenvironment may be an essential prerequisite for axonal regeneration and remyelination. Incorporation of specific essential vascular growth and differentiation factors in peripheral nerve grafts/conduits that facilitate BNB formation is an important consideration with translational potential to the clinic guided by the human BNB transcriptome.

Similarly, this study also provides essential information on the possible determinants of leukocyte trafficking during normal immunosurveillance and the biological networks that may be implicated in peripheral nerve innate and adaptive immune responses. This knowledge could provide insights into understanding the mechanisms of human BNB response to injury, microbial entry from the bloodstream into peripheral nerves and response to viral infections. Our work should also guide studies designed to understand the interactions between the systemic immune compartment and peripheral nerves relevant to understanding the pathogenesis and targeted treatment of peripheral nerve-restricted autoimmune disorders such as Guillain-Barré syndrome and chronic inflammatory demyelinating polyradiculoneuropathy. It is hoped that the deduced human BNB transcriptome and its associated networks and subnetworks will stimulate hypothesis-driven mechanistic studies that would significantly increase our understanding of specialized restrictive barrier-forming human microvascular endothelium.

## Materials and Methods

### Human sural nerve laser-capture microdissection

OCT-cryopreserved sural nerve biopsies stored at −80 °C from 4 adults (2 men and 2 women) with histologically normal peripheral nerves defined as no visually detectable structural abnormalities on routine light microscopy with no known history of systemic or neurological disorders were obtained from the Shin J Oh Muscle and Nerve Histopathological Laboratory, University of Alabama at Birmingham. Informed consent for clinical and research purposes was obtained from every patient undergoing biopsy. The study was approved by the University of Alabama at Birmingham Institutional Review Board, with an exemption obtained to use archived pathological specimens for research (Protocol Number X140321012). All specimen handling and experiments were performed in accordance with relevant guidelines and regulations as stipulated by the University of Alabama at Birmingham. To reduce the impact of RNase activity, RNase-free reagents were utilized whenever available, and all materials and surfaces including tissue slides, cryotome blades and blade holder were cleaned with RNaseZap (Invitrogen) and 70% ethanol prior to sectioning.

20 μm thick axial cryostat sections were transferred to polyethylene naphthalate (PEN) membrane glass slides (Invitrogen catalog number LCM0522) and stored in 50 mL centrifuge tubes at −80 °C for no longer than 10 days. In order to detect endoneurial microvessels, slides were fixed for 2 minutes in 95% ethanol, rinsed twice for 30 seconds in RNase-free phosphate buffered saline (RF-PBS; Ambion), and stained with 500 μL of 100 μg/mL FITC conjugated Ulex Europaeus Agglutinin (UEA)-1 (UEA-1 FITC, Sigma-Aldrich; St. Louis, MO) in RF-PBS for 5 minutes. Slides were washed five times for 10 seconds each in RF-PBS and dehydrated in 70% ethanol for 30 seconds, 95% ethanol for 30 seconds, 100% ethanol for 1 minute and xylene (≥99% pure) for 5 minutes, adapting a protocol used for cerebral microvessels^56^.

UEA-1 FITC-positive endoneurial microvessels were imaged, annotated, and captured using the Arcturus XT system equipped with CapSure Macro laser-capture microdissection [LCM] Caps (Invitrogen catalog number LCM0211). Epineurial and perineurial macrovessels were excluded. Capture was performed using the following infrared (IR) settings: IR spot spacing = 80; IR spot diameter = 6; IR spot power = 80; IR spot duration = 50; IR spot/cut length = 500; tab length = 0; with ultraviolet (UV) cut length = 1500; and UV cutting speed = 1000. Additional xylene dehydration was required to reduce high background fluorescence on some slides prior to annotation and capture. Following capture, each cap was inspected for inappropriately captured tissue. Large extraneous areas were gently removed with tape, while smaller areas were ablated with the laser. Caps were placed in a 0.5 mL microcentrifuge tube with 50 μL RNA extraction buffer, inverted, and stored on dry ice or at −80 °C until RNA isolation. Unless otherwise stated, all reagents were from Thermo Fisher Scientific (Waltham, MA).

Similarly, 20 μm thick axial cryostat sections were also transferred to polyphenylene sulfide (PPS) membrane slides (Leica Microsystems; Buffalo Grove, IL) for LCM using a Leica Microsystems LMD6 scope and stored at −80 °C for no longer than 10 days. To identify endoneurial microvessels, slides were fixed for 2 minutes in 70% ethanol, and washed for 30 and 60 seconds in RF-PBS. Slides were stained 350 μL of 40 μg/mL UEA-1 FITC in RF-PBS for 10 minutes, washed for 30 and 60 seconds in RF-PBS, dehydrated for 2 minutes in 70% ethanol followed by 30 seconds in 100% ethanol, and air dried for 5 minutes. Endoneurial microvessels were microdissected using the Leica system using the “Move and Cut” mode over a 30 min period according to the manufacturer’s instructions, excluding epineurial and perineurial macrovessels. Laser alignment was tested prior to capture for each slide used. The LMD6 system UV (355 nm) laser uses a pulse frequency of 80 Hz, pulse length of < 4 ns and maximum pulse intensity of 70 μJ, with dissected material dropping into the collection tube cap by gravity. Sample humidity levels were controlled by limiting drying times and excessive static was alleviated by dipping slides in 70% alcohol. Following capture, 50 μL of Qiazol (Qiagen; Hilden, Germany) was added to well of cap, the tube was closed and incubated inverted at room temperature for 5 minutes, and stored on dry ice or −80 °C until RNA isolation.

### RNA isolation

RNA was isolated from endoneurial microvessels dissected with the Arcturus XT system using the Arcturus Pico Pure Isolation Kit (Thermo Fisher Scientific), according to the manufacturer’s directions. Caps were heated at 42 °C for 35 minutes (using Pico Pure extraction buffer (XB)) for 15 minutes with membrane removal using RNaseZap-treated forceps and placed in bottom of tube in XB for additional 20 minutes. Following RNA binding to the column, an on-column DNase digestion for 15 minutes using 80 μL DNase Mixture (Qiagen RNase-free DNase Set, catalog number 79254) was performed to remove contaminating genomic DNA. RNA was eluted using 20 μL RNase-free water supplied with the kit. RNA from endoneurial microvessels dissected with the Leica system was extracted using the miRNeasy kit (Qiagen, Hilden, Germany, catalog 217084), according to the manufacturer’s instructions. On-column DNase digestion was also performed to remove contaminating genomic DNA using the RNase-free DNase Set (Qiagen, catalog79254). Isopropanol-containing RNA was eluted using RNase-free water. Extracted RNA was stored at −80 °C prior to further use.

### Primary human endoneurial endothelial cell culture and RNA isolation

Primary human endoneurial endothelial cells (pHEndECs) were previously isolated and purified from the sciatic nerves of a decedent 38 year old woman with no known history of systemic or neurological disease via endoneurial stripping, and sequential enzymatic digestion and density centrifugation, and cultured as published^7^. pHEndECs were expanded on rat-tail collagen-coated Coring CellBIND^®^ flasks or Petri dishes in freshly prepared growth medium consisting of RPMI-1640, 10% NuSerum, 10% fetal bovine serum, 1% vitamin solution, 1% non-essential amino acid solution, 1% sodium pyruvate, 1% penicillin-streptomycin, 2 mM L-glutamine, 10 mM HEPES buffer, and enriched with 50 μg/mL endothelial cell growth supplement, 1 ng/mL human recombinant basic fibroblast growth factor and 10 U/mL heparin, from the same vendors. pHEndECs were passaged at 80–90% confluence, and allowed to grow to complete confluence at early- (P3) and late- (P8) passages before total RNA was extracted 24 hours later using TRIzol reagent according to the manufacturer’s instructions, as previously published^7,25,27^. Extracted RNA was stored at −80 °C prior to further use.

### RNA-Sequencing

RNA yield and quality were determined on an Agilent 2100 BioAnalyzer using the Pico Chip (Santa Clara, CA). RNA obtained from a minimum of 200 laser-capture microdissected endoneurial microvessels per adult nerve were pooled and concentrated on a Savant SpeedVac (Thermo Fisher Scientific; Waltham, MA) or used as extracted from P3 and P8 pHEndECs. cDNA libraries were generated using the SMARTer Stranded Total RNA-Seq Kit - Pico Input Mammalian (Clontech, catalog 635005) and ribosomal cDNA was depleted prior to the final amplification step as per the manufacturer’s instructions. High Sensitivity DNA electropherogram traces were captured on the BioAnalyzer to confirm the quality of the RNA-Seq Libraries. The final libraries were quantified by quantitative PCR (Kapa Biosystems, Inc. Woburn, MA). Equal nanomolar amounts of libraries were mixed prior to loading on to the sequencer.

Next Generation Sequencing was performed by the UAB Heflin Center Genomics Core using the Illumina NextSeq. 500 (100 Gb/flowcell) Sequencing Platform setup to capture 75 base pairs (bp), paired end sequences (for endoneurial microvessels) and 50 bp, paired end sequences (for pHEndECs), as per the manufacturer’s standard protocols (Illumina Inc. San Diego CA). Quality (Q) Scores (prediction of base calling error probability from the sequencer) were determined for each base pair from each sample (in duplicate). Sequence alignment was performed using STAR program referenced to Genome Reference Consortium Human Reference 37 (hg19). Transcript annotation was performed using PANTHER (http://www.pantherdb.org/)^57^.

### Transcript analyses

Transcript quantification for the P3 and P8 pHEndECs, and the laser-capture microdissected endoneurial microvessels from four normal adult sural nerve biopsies was performed using Fragments Per Kilobase of transcript per Million (FPKM). FPKMs were generated using Cufflinks and Cuffdiff software programs^58^. Guided by currently known transcript or protein expression at the human BNB *in vitro* or *in situ* ^4,7,17,24,27,28,30,32,35,37,59^, a detection threshold of FPKM >0.1 was set to identify expressed transcripts. Transcripts with FPKM ≤0.1 were defined as no expression and transcripts with zero FPKM in all samples were removed.

The *in vitro* BNB (IVBNB) was defined as transcripts expressed by both P3 and P8 pHEndECs. The *in situ* BNB (ISBNB) was defined as transcripts expressed in at least 3 out of 4 adult sural nerve biopsy laser-capture microdissected endoneurial microvessel samples. The human BNB transcriptome was defined as transcripts expressed by both the IVBNB and ISBNB. These common transcripts should exclude pericyte, Schwann cell, fibroblast, leukocyte and axonal transcripts that may contaminate early passage pHEndECs or laser-capture microdissected endoneurial microvessels. To identify possible transcripts expressed by the human BNB *in situ* that may be lost with pHEndEC culture, transcripts expressed by the ISBNB and P3 pHEndECs were also identified. The average IVBNB and ISBNB FPKMs for each transcript was used for hierarchical clustering. Transcript overlaps between the P3 and P8 pHEndECs, and the IVBNB and ISBNB were generated using custom scripts. Cluster 3.0 and JAVA Treeview software packages were used for hierarchical clustering and heatmap generation respectively^60,61^.

The human BNB transcriptome functional classification was performed using PANTHER. This program performs pathway enrichment analyses referenced to GO ontology and utilizes Fisher’s one-tailed test (also known as cumulative hypergeometric probability) with its specific multiple testing correction algorithm (g:SCS) to determine the probability that genes associated by a specific network/subnetwork occur by random chance. Lower p-values are generally indicative of higher odds that the assigned association is important and reveals relevant biological information. Statistical significance is set at p ≤ 0.05. Pie charts and bar histograms were generated to demonstrate BNB biological function, cellular component and protein class GO-terms distribution and quantity^62^. Lists of transporters, junctional complex components and chemokines/chemokine receptors expressed by the human BNB were generated by referencing PANTHER generated functional classification lists based on gene ID against the database of all identified human BNB transcripts and recently published references on these gene subfamilies to ensure completeness^46,63–69^.

### Transcript expression validation

Validation of transcript expression by the deduced human BNB was performed *in situ* by indirect fluorescent immunohistochemistry on 10 μm thick cryostat sections of a histologically normal cryopreserved sural nerve biopsy from a 44 year old woman using an array of 31 adhesion, cell membrane, chemokine receptor, cytoskeletal, junctional complex (including adherens, gap and tight junctions and their adapters), secreted and transporter proteins with mean transcript FPKM values varying between 0.8 and 750.5 *in situ* and 0.4 and 3012.4 *in vitro* (Table 1).

**Table 1:** Human Blood-Nerve Barrier Transcriptome validation antibodies.

Gene Symbol	Protein Name	*In Vitro* FPKM	*In Situ* FPKM	Antibody Source	Catalog Number	Host/Isotype	Final Concentration
*ABCA8*	Lipid transporter	0.4	119.3	Life Technologies	PA5-60866	Rabbit polyclonal IgG	2 µg/mL
*ABCB1*	P-glycoprotein	2.3	23.0	Life Technologies	MA5-13854	Mouse IgG1	4 µg/mL
*ACTG1*	Gamma 1 actin	3012.4	242.7	Life Technologies	PA5-13467	Rabbit polyclonal IgG	40 µg/mL
*AQP1*	Aquaporin 1	1.68	29.0	Santa Cruz Biotechnology	sc-25287	Mouse IgG1 (kappa light chain)	4 µg/mL
*CALD1*	Caldesmon 1	140.6	750.5	Abcam	ab68878	Rabbit polyclonal IgG	1 µg/mL
*CAV1*	Caveolin 1	681.7	43.0	Santa Cruz Biotechnology	sc-53564	Mouse IgG2b (kappa light chain)	4 µg/mL
*CD44*	Cell surface adhesion glycoprotein	120.0	50.1	Santa Cruz Biotechnology	sc-7297	Mouse IgG1 (kappa light chain)	4 µg/mL
*CD63*	Tetraspanin family member glycoprotein	1132.9	33.1	Santa Cruz Biotechnology	sc-5275	Mouse IgG2a (kappa light chain)	4 µg/mL
*CDH5*	Cadherin 5	340.1	19.1	Santa Cruz Biotechnology	sc-9989	Mouse IgG1 (kappa light chain)	4 µg/mL
*CDH6*	Cadherin 6	1.8	7.2	Life Technologies	MA1-06305	Mouse IgG1	20 µg/mL
*CLDN4*	Claudin 4	0.9	2.5	Life Technologies	36-4800	Rabbit polyclonal IgG	2.5 µg/mL
*CLDN5*	Claudin 5	362.1	17.7	Life Technologies	35-2500	Mouse IgG1	10 µg/mL
*CTGF*	Connective tissue growth factor	709.0	44.6	Santa Cruz Biotechnology	sc-101586	Mouse IgG1	2 µg/mL
*CTNNA1*	Alpha 1 catenin	158.6	218.7	Life Technologies	13-9700	Mouse IgG1 (kappa light chain)	2 µg/mL
*CXCR4*	CXC chemokine receptor 4	263.8	3.4	Santa Cruz Biotechnology	sc-53534	Mouse IgG1 (kappa light chain)	4 µg/mL
*ESAM*	Endothelial cell adhesion molecule	199.1	4.6	R&D Systems	MAB4204	Mouse IgG2b	10 µg/mL
*FLNA*	Filamin A	795.9	553.5	Santa Cruz Biotechnology	sc-17749	Mouse IgG2a (kappa light chain)	4 µg/mL
*GJA1*	Gap junction protein alpha 1	170.4	7.2	Life Technologies	13-8300	Mouse IgG1	10 µg/mL
*LIN7A*	Lin 7A, crumbs cell polarity complex	0.4	0.8	Life Technologies	PA5-30871	Rabbit polyclonal IgG	20 µg/mL
*MPDZ*	Multiple PDZ domain crumbs cell polarity complex component	18.9	43.8	Sigma-Aldrich	HPA020255	Rabbit polyclonal IgG	2 µg/mL
*MYO10*	Myosin X	29.8	15.0	Life Technologies	PA5-55019	Rabbit polyclonal IgG	6 µg/mL
*PCDH1*	Protocadherin 1	61.7	15.0	Life Technologies	PA5-35091	Rabbit polyclonal IgG	9.4 µg/mL
*SLC1A1*	Glutamate transporter	7.3	2.5	Cell Signaling Technology	14501	Rabbit polyclonal IgG	320 µg/mL
*SLC2A1*	Glucose transporter 1	54.4	105.4	Santa Cruz Biotechnology	sc-377228	Mouse IgG1 (kappa light chain)	4 µg/mL
*SLC3A2*	L-type amino acid transporter	107.5	53.4	Santa Cruz Biotechnology	sc-376815	Mouse IgG1 (kappa light chain)	4 µg/mL
*SLC5A6*	Sodium-dependent multivitamin transporter	11.4	1.5	Sigma-Aldrich	HPA036958	Rabbit polyclonal IgG	4 μg/mL
*SLC16A1*	Monocarboxylate transporter 1	36.1	1.7	Santa Cruz Biotechnology	sc-365501	Mouse IgG1 (kappa light chain)	4 µg/mL
*SLC19A2*	Thiamine transporter	2.4	2.6	Life Technologies	PA5-53456	Rabbit polyclonal IgG	2 µg/mL
*TJP1*	Zona occludens-1	32.6	88.4	Life Technologies	61-7300	Rabbit polyclonal IgG	2.5 µg/mL
*VEZT*	Vezatin	28.1	11.9	Life Technologies	PA5-52115	Rabbit polyclonal IgG	6 µg/mL
*ZYX*	Zyxin	201.1	35.8	Life Technologies	PA1-25162	Rabbit polyclonal IgG	133 µg/mL

Consecutive 10 μmthick axial cryostat sections were fixed in acetone at −20 °C for 5 minutes, washed with 1X Phosphate Buffered Saline (PBS) and air-dried at room temperature for 5 minutes prior to blocking with 10% normal goat serum (NGS) in 1X PBS for 30 minutes. Without washing, each slide was incubated with a single primary antibody diluted in 2% NGS in 1X PBS for 1 hour at room temperature. Following washing, slides were incubated with either goat anti-mouse IgG (H + L) Alexa Fluor^®^ 594 conjugate (Catalog # A-11005, Life technologies, Carlsbad, CA: 4 μg/mL) or goat anti-rabbit IgG (H + L) Alexa Fluor^®^ 594 conjugate (Catalog #A-11037, Life technologies: 4 μg/mL) with UEA-1-FITC (Catalog # L9006, Sigma-Aldrich, 10 μg/mL) in 2% NGS in 1X PBS for 1 hour at room temperature in the dark. Following a final wash, all sections were stained with 0.45 μM 4, 6-diamidino-2-phenylindole (DAPI) for 5 minutes to detect nuclei and mounted with ProLong^®^ Gold antifade mounting medium (Life technologies, Catalog # P36934). Digital photomicrographs were taken using an Eclipse Ci-S Upright epifluorescent microscope with a D5-Qi2 camera (Nikon Instruments Inc., Melville, NY) and merged using the Nikon Elements software.

### Data and materials availability

The human blood-nerve barrier transcriptome data (including Supplementary Data file S3) have been deposited in National Center for Biotechnology Information's Gene Expression Omnibus and are accessible through GEO Series accession number GSE107574 (https://www.ncbi.nlm.nih.gov/geo/query/acc.cgi?acc=GSE107574). pHEndECs may be obtained through an MTA. Detailed isolation protocols have been previously published.

## Electronic supplementary material

Supplementary information

Dataset 1

Dataset 2

Dataset 3

Dataset 4

Dataset 5

Dataset 6

Dataset 7

Dataset 8
