# Supplementary material for: The Human Blood-Nerve Barrier Transcriptome
Source: Sci Rep. 2017 Dec 12;7:17477. doi: 10.1038/s41598-017-17475-y (PMC5727190; doi:10.1038/s41598-017-17475-y)
Supplement: Supplementary file 1 — Supplementary information [file 41598_2017_17475_MOESM1_ESM.pdf]

The Human Blood-Nerve Barrier Transcriptome

Steven P. Palladino, E. Scott Helton, Preti Jain, Chaoling Dong, Michael R. Crowley, David K. Crossman, Eroboghene E. Ubogu

Supplementary Materials (Key):

Data files S1-S6: Header description is as follows: **Signf-p-value**: Significant enrichment p-value; **T**: Total number of genes associated to functional term; **Q**: Number of genes in input list. This varies when list is ordered and optimum list length is detected ; **Q&T**: Number of genes in the list associated to functional term; **Q&T/Q**: Fraction of genes in list with the functional term (precision); **Q&T/T**: Fraction of all functional genes detected in the list (recall); **term ID**: term identification; **t type**: term type or GO domain (BP= Biological Process, CC= Cellular Component, MF=Molecular Function), **t group**: term group; **t name**: term name; **t depth**: term depth; **Q&T list**: list of genes in the list associated to functional term.
